# Supra- and sub-gingival instrumentation of periodontitis with the adjunctive treatment of a chloramine – a one-year randomized clinical trial study

**DOI:** 10.1080/00016357.2023.2281486

**Published:** 2024-03-22

**Authors:** Viveca Wallin-Bengtsson, Ulrica Scherdin-Almhöjd, Ann-Marie Roos-Jansåker

**Affiliations:** a Department of Oral Health, Faculty of Oral Health Science, Kristianstad University, Kristianstad, Sweden; b Department of Cariology, Institute of Odontology, Sahlgrenska Academy, Gothenburg, Sweden; c Department of Periodontology, Faculty of Odontology, Malmö University, Malmö, Sweden; d Department of Periodontology, Blekinge Hospital, Karlskrona, Sweden

**Keywords:** Chloramine, 0HIP, supra- and subgingival instrumentation

## Abstract

Periodontitis is a bacterial-induced disease and for this reason controlling the microbiota is a necessity. Therapy includes self-performed daily oral hygiene in combination with supra- and sub-gingival instrumentation. An adjunctive antimicrobial agent may improve the outcome.

**Aims:**

To assess whether a chloramine (Perisolv^®)^ has an adjunctive effect to non-surgical periodontal therapy and whether non-surgical periodontal therapy affects quality of life.

**Material and Methods:**

Thirty-eight patients were randomized to a test or a control group. Clinical indices were performed at baseline and at three and twelve months. In the test group, Perisolv® was applied initially and after the sub-gingival instrumentation in pathological pockets. Oral health-related quality of life was measured with the Oral Health Impact Profile (OHIP) instrument at baseline and twelve months.

**Results:**

In both groups, an initial probing pocket depth (PPD) of > 4mm and bleeding on probing (BOP) were statistically reduced (*p* < 0.002 and *p* < 0.002 respectively) at twelve months and after adjustment for Bonferroni. There were no significant differences between the test and the control group in terms of the number of PPD, BOP or plaque index, or in the mean OHIP score.

**Conclusions:**

Chloramine did not have an adjunctive effect, but the overall therapy was significantly efficacious both clinically and in terms of quality of life.

**Trial registration:**

Registered at www.clinicaltrials.gov: NCT05757921.

## Introduction

Periodontitis is a result of a polymicrobial community of key bacteria in the oral biofilm that lead to an inflammatory response by the periodontium [1]. Furthermore, it is a major cause of tooth loss [2]. Globally, severe periodontitis affects about 19% of the world’s population [3] making it one of the six most prevalent diseases in the world [4]. Although bacteria are necessary for the initiation of the disease, this is not sufficient to progress the disease unless there is an inflammatory response in a susceptible host. The receptivity may depend on immune deficiencies, immune regulatory defects or environmental factors such as smoking, diet, stress, or epigenetic changes [5–8].

Dental plaque is defined as a microbial biofilm consisting of pathogenic bacteria embedded in a matrix of carbohydrate polymers developing on the tooth surface [1]. Furthermore, there are over 700 different species of pathogenic bacteria reported in the oral cavity [9]. When dental plaque mineralises, calculus composed of calcium phosphate salts forms, and it is covered by a biofilm [10]. Some bacteria have been associated with oral health, but, during a microbiological shift (dysbiosis), disease may appear [11]. There is wide consensus that periodontitis is a biofilm-associated disease [12] since there is an interaction between the oral micro-organisms in the biofilm and the immune-cell response [13].

It is believed that self-performed oral hygiene plays an important role in the prevention and treatment of periodontal disease [14,15]. To maintain gingival health, oral hygiene skills, a minimum of once every 48 h, are needed [16]. However, it has been shown that most people do not clean their teeth efficiently enough, with too little time spent on brushing, on average 40 s, thereby leaving plaque on the tooth surface [17]. For this reason, brushing twice daily is a better habit [18], for a period of two minutes [19]. The new clinical practice guideline highlights the importance of behavioral changes, including the successful removal of the supragingival biofilm [20].

When calculus has formed on the root surface, it may interfere with oral hygiene procedures and a pathogenic bio-film will grow. For adequate periodontal treatment, the calculus must be removed [21].

From the European Federation of Periodontology (EFP) clinical practice guidelines, the “golden standard” for treating periodontitis is divided into three steps [20]. The first step includes adequate self-performed oral hygiene and supra-gingival professional mechanical plaque removal, while the second step is the professional elimination of the sub-gingival biofilm and calculus with or without adjunctive therapies. Finally, the third step is optional and includes repeated sub-gingival instrumentation with or without an adjunctive or surgical intervention. Hand- or power-driven instruments remove the calculus. The choice of method either alone or in combination should be based on operator skills and patient agreement [20,22]. The two techniques, or a combination of both, have shown residual calculus on the root surfaces post treatment [23]. The re-colonization of pathogens and thereby the recurrence of the disease is common [24]. Because of this, the use of an antimicrobial therapy in combination with sub-gingival instrumentation is suggested [25]. In general, treatments published within step two include antibiotics, chlorhexidine, and chloramines [20].

Studies have shown some extended effects on clinical parameters using local [26,27] or systemic antibiotics [28,29]. However, from a global perspective, antibiotics should only be used in aggressive forms of periodontitis, considering the fast-developing antibiotic resistance [30,31].

The antiseptic agent, chlorhexidine, has shown inconclusive results and has not been proven to give an adjunctive effect to sub-gingival instrumentation [26,32]. However, chlorhexidine rinse may be considered in some specific cases for a limited time [20,33–35]. There are other adjunctive therapies, such as systemic sub-antimicrobial-dose doxycycline which is recommended “not to use” by the European Federation of Periodontology (EFP) in 2020. Further adjunctive application of lasers to subgingival instrumentation (wavelength range of 2780– 2940 nm and of 810–980 nm) has shown insufficient evidence to be recommended. No benefits with the adjunctive application of antimicrobial photodynamic therapy have been observed [25].

Chloramines are another antimicrobial adjunctive therapy [36]. Commercial chloramines such as Perisolv® (earlier Perio+) were introduced on the market in 2009 by Orasolv AB and were recently redistributed by Regedent AG with the intention of cleaning the dental pockets in patients diagnosed with periodontitis, peri-implantitis or mucositis. The mechanism of the formed chloramines is not yet fully understood, but the active chlorine components are antibacterial and appear to disturb the biofilm [37]. Other in-vitro studies have shown increased cell survival in periodontal ligament cells on root surfaces [38]. There are still a limited number of randomized clinical studies, but the additive effect in patients with peri-implantitis show that Perisolv® is equivalent to mechanical debridement at three months [39] and, with ultrasonic debridement, demonstrate a PPD reduction of 1 mm, at twelve months, in residual pockets [40]. From a six-month study, the removal of the sub-gingival biofilm in deep periodontal pockets may be improved in conjunction with minimally invasive non-surgical therapy (MINST) [41].

The oral health-related quality of life (OHQoL) may improve after receiving proper dental care [42]. Studies have shown that the number and location of remaining natural teeth, the number of occluding pairs [43,44] and periodontal disease [45] affect quality of life.

The aims of this study were to assess whether a chloramine (Perisolv®) has an adjunctive effect to non-surgical periodontal therapy and whether non-surgical periodontal therapy affects quality of life.

## Material and methods

Prior to the study, two clinicians (VWN, AMRJ) were trained and calibrated in measuring and recording clinical indices. The Regional Ethics Review Board at Lund University, Sweden, approved the protocol for the study (LU 2011/462) and the World Medical Association Declaration of Helsinki was followed. The study is registered under www.clinicaltrials.gov (study no. NCT05757921).

The primary outcome was probing pocket depth reduction. From the power analysis 32 individuals were minimum to reach a clinically significant difference at 0.5 mm in probing pocket reduction between the groups with alpha 0.05 and power 80%. Thirty-eight patients with periodontitis were consecutively invited to participate in the study at the Department of Periodontology, Kristianstad, Sweden. After being given written information, all the patients signed a consent form. The enrolment occurred, between September 2011, until September 2012. The different treatments were blinded to the two examiners (VWB, AMRJ) who further recorded the measurements and clinical indices at baseline, three months, and twelve months. The supra- and sub-gingival instrumentation was carried out by a non-blinded dental hygienist (BLN) who also recorded the plaque index (PI) and gave oral hygiene instructions. This was performed during the treatment phase and at the supportive therapy at six and nine months.

### Selection of patients

Adult patients diagnosed with periodontitis were consecutively invited to participate.

#### Inclusion

- Adult patients over the age of 18 years
- Minimum 10 teeth
- Probing pocket depth (PPD) of ≥ 5 mm at ≥ 4 sites with additional bleeding and probing (BOP)

#### Exclusion

- Uncontrolled diabetes (HbA1c > 7)
- In need of antibiotic prophylaxis
- On medication with corticosteroids or medication with the side-effect of gingival overgrowth
- Systemic antibiotics three months prior to the initiation of the study

#### Assessment of patients

At baseline, a clinical and radiographic examination was performed by either of the two examiners, specialists in periodontology (VWB, AMRJ). The following recordings were performed on all teeth at six sites and registered at four sites:

- Probing pocket depth (PPD) to the nearest mm
- Bleeding on probing (BOP) after 30 seconds
- Pus (P) present or non-present
- Plaque index (PI) visible signs of plaque after disclosure Diaplac^TM^

### Randomization of patients and treatment groups

Once the entry criteria had been confirmed, the patients were included in the study and assigned a patient number. The patients were randomly assigned to one of the two different treatment groups according to pre-defined randomization. The assignment of test or control was carried out by randomly placing cards in sealed envelopes with unique numbers. The assigned treatment was performed on the patient by the treating dental hygienist after the envelopes were opened.

Test group: In all pathological pockets of ≥ 4 mm, Perisolv® was applied initially. Supra- and sub-gingival instrumentation using piezo electronic ultrasound, Electro Medical Systems (EMS), in combination with conventional periodontal hand instruments, was performed. After the final treatment, Perisolv was once again administered in the pocket.

Control group: Supra- and sub-gingival instrumentation using piezo electronic ultrasound, EMS, in combination with conventional periodontal hand instruments was performed.

Extracted teeth and teeth that were judged to require periodontal surgery were seen as dropouts.

### Questionnaire

Oral health-related quality of life (OHQoL) was measured in all patients with an Oral Health Impact Profile (OHIP) using a total of 14 questions from the original OHIP-49 questionnaire at baseline and three and twelve months respectively. From our perspective, the most relevant questions regarding this study were chosen. Questions did not follow the setup in the OHIP-14 by Slade, G. D. (1997) completely, but they were all derived from the OHIP-49, which is regarded as a reliable and valid instrument for the detailed measurement of the social impact of oral disorders [46].

The questions were divided into seven domains. Each domain contained one to two questions on a four-level scale ranging from “never” (score 0), “hardly ever” (score 1), “occasionally” (score 2), “fairly often” (score 3) and “very often” (score 4). OHQoL was expressed as the sum of the scores for the 14 questions (OHIP score, maximum 56). The mean values for patients in the test group, the control group and all patients combined were calculated. The prevalence of different items was defined as experiencing the item occasionally, fairly often or very often. Patients who did not answer all the OHIP questions were excluded from the analysis of the total OHIP score. Concerning one and each of every single question, all the given answers were reported irrespective of whether all the OHIP questions were filled in.

### Schedule of visits

The visits were scheduled as follows:

*Baseline*: Clinical (periodontal indices) and radiographic examination (VWB, AMRJ), OHIP

*Week 1*: PI and oral hygiene instruction, supra- and sub-gingival instrumentation (test or control), polishing (BLN)

*Week 2*: PI and oral hygiene instruction, supra- and sub-gingival instrumentation (test or control), polishing (BLN)

*Week 3*: PI and oral hygiene instruction, supra- and sub-gingival instrumentation (test or control), polishing (BLN)

*Week 4*: PI and oral hygiene instruction, supra- and sub-gingival instrumentation (test or control), polishing (BLN)

*Week 6-8:* Oral hygiene check

*Three months after the initial treatment:* Periodontal indices and oral hygiene reinforcement (VWB, AMRJ), supportive therapy (BLN), OHIP

Six months after the initial treatment: Supportive therapy (BLN)

*Twelve months after the initial treatment*: Clinical and radiographic examination (VWB, AMRJ), OHIP

### Statistics

The data were analyzed using descriptive and comparative statistics. Mean values, standard deviation (SD) and frequency distributions are given. Independent t-tests (equal variance not assumed), a paired t-test and a one-way ANOVA test were used to compare inter- and intra-group differences. The non-parametric McNemar test was used for categorical variables. Statistical significance was considered at *p* ≤ 0.05 and significant values have been adjusted for the Bonferroni correction for multiple tests. The IBM SPSS version 27.0 statistical software package (SPSS Inc., Armonk, NY, USA) for personal computers was used in the analyses.

## Results

### Clinical indices

In the test and control group, 20 and 18 individuals respectively were initially included. For demographic data, see (Supplementary Table 1). There were no significant differences between the con-trol and test groups in the number of teeth, number of sites and number of the different initial PPD, BOP or PI, independent of time point (Supplementary Tables 2-4).

In BOP there were significant reductions, independent of time point, within both the test and the control group, and adjusted for Bonferroni (Supplementary Tables 5-6, Figure 1).

Concerning the PI, a reduction was shown in both groups, although not significant in the test group, independent of time point. Significant reduction was shown in the control group at twelve months and remained significant after adjustment for Bonferroni (Supplementary Tables 5-6, Figure 2).

Within both groups, there were significant reductions in PPD≥ 6 mm, and adjusted for Bonferroni, at three and twelve months (Supplementary Tables 5-6).

### Oral health Impact Profile (OHIP)

There were no statistical differences regarding the OHIP score between the test group and the control group, independent of time point. Over time, the mean OHIP score was reduced in both the test and the control group, but the reduction was not significant. There was only a significant reduction in the mean OHIP score for all patients combined from baseline to twelve months, with a reduction in the OHIP score from 13.6 to 11.6 (*p* = 0.023), the significance remained after adjustment for Bonferroni. The prevalence of different problems never changed statistically after non-surgical therapy (Supplementary Table 7).

## Discussion

The basic aim of non-surgical treatment is to reduce the inflammatory burden to prevent further disease progression, targeting infection control and pocket reduction [47]. In this study, a chloramine was tested as an adjunct to supra- and sub-gingival instrumentation. The main findings in both groups were significant improvements in the clinical indices such as the reduction in probing pocket depths (PPD) ≥ 6 mm and gingival inflammation (BOP). Perisolv® as an adjunct made no difference in the reduction of PPD or BOP. Bleeding is an indicator of inflammation and pathology [48]. According to Chapple et al. (2018), a clinical case of health is considered when the BOP is < 10%, which, in our study, is close to the BOP at twelve months within both groups [49]. In the control group, the BOP dropped from 46% to 11%, while it fell from 58% to 13% in the test group. The larger drop in the test group between baseline and twelve months indicates that chloramine may have had an antimicrobial effect [38] and perhaps also a healing effect [50].

Numerous studies have shown successful results in both medium-deep and deep pockets with supra- and sub-gingival instrumentation, and it is regarded as the gold standard in periodontitis [25]. In both groups, the mean number of deep pockets of ≥ 6 mm was statistically reduced. After twelve months, the number of PPD ≥ 6 mm was reduced from 24.4 to 5.1 in the test group. The corresponding figures for the control group was a reduction of PPD ≥ 6 mm from 19.7 to 4.1. This agrees to some extent with a review and meta-analysis by Citterio et al. 2022, who reported that a PPD of ≥ 6 mm decreased from an initial 24.6 to 7.33 at twelve months [51].

In spite of a statistical reduction in PPD≥ 6 mm and BOP within each group, there were no significant differences between the test and control groups regarding the reduction in PPD, BOP and PI. The hygienist was technically skilled in supra- and sub-gingival instrumentation and had a good knowledge of motivating and educating patients in good oral hygiene habits. So, because of meticulous supra- and sub-gingival instrumentation, the adjunctive chloramine may have been redundant [39]. According to the national guidelines in Sweden, administration of chemical antimicrobials in the probing pockets as an adjunct to mechanical infection control is not endorsed [52].

It is important not only to focus on the clinical outcomes. A patient-based focus is crucial to understand the impact of the treatment. In this study, a questionnaire comprising OHIP questions picked from the OHIP 49 by Slade and Spencer (1994) was used [53]. The maximum result for OHIP gave a score of 56, where the mean for the test and control groups differed marginally at baseline, with scores of 13.4 and 14.0 respectively. The twelve-month scores were significantly reduced in all patients combined, which indicates an increase in patient quality of life (OHQoL) after non-surgical treatment.

As our instrument for measuring OHQoL consists of questions from the OHIP 49 instrument, where all the questions were validated per se but differed from the original OHIP-14 instrument, it is difficult to make comparisons with other studies. However, in another study, the total OHIP-14 score was significantly reduced from baseline to one month after subgingival instrumentation, indicating that the OHQoL had improved [54].

It is remarkable that the prevalence of the questions regarding “food catching”, “sensitive teeth”, “sore jaw” and “worried” was high at baseline, ranging from 39%-89%, and persisted after non-surgical therapy. It should be noted that the prevalence was determined as experiencing the specific problem occasionally, fairly often or very often. Consequently, the severity of the different items might have been reduced but may still be prevalent. Problems with food catching and sensitive teeth were large and, for this reason, more effort to reduce symptoms from sensitive teeth and sore jaws should be considered. We strongly underline the importance of including oral health-related quality of life in non-surgical periodontal therapy. The absence of differences in the mean OHIP score, as well as the prevalence of problems in the different questions between the test and control groups, indicates that the patients perceived no difference regardless of whether an adjunct of chloramine was included in the supra- and sub-gingival instrumentation.

The strength of this study is that the dental professionals constituted a well-established and experienced team.

## Conclusions

In this study, we showed improvements in all the clinical indices with the supra- and sub-gingival instrumentation of periodontitis independent of an adjunctive chloramine.

## Supplementary Material

Supra- and sub-gingival instrumentation of periodontitis with the adjunctive treatment of a chloramine – a one-year randomized clinical trial study

Supra- and sub-gingival instrumentation of periodontitis with the adjunctive treatment of a chloramine – a one-year randomized clinical trial study

## Data Availability

The data supporting the findings in this study are available in response to a reasonable request.
