# Supplementary material for: Supra- and sub-gingival instrumentation of periodontitis with the adjunctive treatment of a chloramine – a one-year randomized clinical trial study
Source: Acta Odontol Scand. 2024 Mar 22;83:40288. doi: 10.1080/00016357.2023.2281486 (PMC11302644; doi:10.1080/00016357.2023.2281486)
Supplement: Supra- and sub-gingival instrumentation of periodontitis with the adjunctive treatment of a chloramine – a one-year randomized clinical trial study [file AOS-83-40288-s1.pdf]

Supplementary material has been published as submitted. It has not been copyedited or typeset by Acta Odontologica Scandinavica.

Table 1. Baseline characteristics and patient demographics

**Characteristic**	Test group (n=20)	Control group (n=18)
**Gender**		
Male	10 (50%)	9 (50%)
Female	10 (50%)	9 (50%)
**Age**		
Mean	54.9	51.8
Median	56.5	52.5
SD	11.4	12.1
Range	32-71	24-69
**Disease**		
Cardiovascular diseases	6 (10%)	2 (11%)
Diabetes	3 (15%)	1 (6%)
**Medication**		
Yes	10 (50%)	4 (33%)
No	10 (50%)	12 (67%)
**Smoker**		
Yes	9 (45%)	7 (39%)
Former	8 (40%)	6 (33%)
No	3 (15%)	5 (28%)
**Smoking years**		
Mean	23.9	21.2
Median	22.5	26.0
SD	11.9	15.4
Range	0-54	0-41

Table 2. Baseline clinical parameters between the groups

	Test group	Control group	Total group	p-value
**Number of teeth**				
(n)	496	457	953	p=0.702
Mean	24.8	25.4	25.1	
Median	25.0	26.0	26.0	
SD	4.8	4.6	4.6	
Range	11-30	12-32	11-32	
**Number of sites**				
(n)	1984	1828	3812	p=0.702
Mean	99.2	101.6	100.3	
Median	100.0	104.0	104.0	
SD	19.1	18.4	18.6	
Range	44-120	48-128	44-128	
**Number of PPD ≤ 3 mm** (n/%)	1013/51	985/54	1998/52	p=0.652
Mean	50.7	54.7	52.6	
Median	51.0	60.0	53.0	
SD	21.1	25.2	22.9	
Range	5-81	7-91	5-91	
**Number of PPD 4 mm** (n/%)	237/12	288/16	525/14	p=0.398
Mean	11.9	16.0	13.8	
Median	11.0	14.5	12.5	
SD	11.0	12.3	11.6	
Range	0-37	0-36	0-37	
**Number of PPD 5 mm** (n/%)	246/12	200/11	446/12	p=0.534
Mean	12.3	11.1	11.7	
Median	14.0	11.0	11.5	
SD	7.9	2.8	6.0	
Range	0-34	6-15	0-34	
**Number of PPD ≥ 6 mm** (n/%)	488/25	355/19	843/22	p=0.388
Mean	24.4	19.7	22.2	
Median	21.5	17.0	17.5	
SD	15.3	14.5	14.9	
Range	6-65	2-59	2-65	
**BOP** (%)				p=0.074
Mean	58.3	46.3	52.3	
Median	57.5	44.5	49.5	
SD	20.7	19.3	20.7	
Range	24-100	22-90	22-100	
**PI** (%)				p=0.638
Mean	47.6	51.4	49.4	
Median	42.5	47.5	46.0	
SD	27.1	22.3	24.7	
Range	12-100	24-100	12-100	

Statistical analysis by two-way ANOVA was used. Statistical significance was determined at $p < 0.05$. Test group= non-surgical treatment with a chloramine as an adjunct. Control group= non-surgical treatment.

Table 3. Three-months clinical parameters between the groups

	Test group	Control group	Total group	p-value
**Number of teeth**				
(n)	454	388	842	p=0.166
Mean	23.9	25.8	24.8	
Median	24.0	26.0	25.5	
SD	4.5	3.3	4.1	
Range	11-30	17-32	11-32	
**Number of sites**				p=0.166
(n)	1816	1552	3368	
Mean	95.6	103.5	99.1	
Median	96.0	104.0	102.0	
SD	18.1	13.2	16.4	
Range	44-120	68-128	44-128	
**Number of PPD ≤ 3 mm** (n/%)	1356/75	1191/77	2547/76	p=0.624
Mean	71.4	79.4	74.9	
Median	80.0	86.0	83.5	
SD	24.1	24.2	24.1	
Range	23-104	25-112	23-112	
**Number of PPD 4 mm** (n/%)	125/7	155/10	280/8	p=0.561
Mean	6.6	10.3	8.2	
Median	4.0	4.0	4.0	
SD	7.8	13.1	10.5	
Range	0-24	0-49	0-49	
**Number of PPD 5 mm** (n/%)	132/7	87/5	219/7	p=0.336
Mean	7.0	5.8	6.4	
Median	6.0	3.0	4.5	
SD	4.3	6.3	5.2	
Range	0-14	0-21	0-21	
**Number of PPD ≥ 6 mm** (n/%)	203/11	119/8	322/10	p=0.282
Mean	10.7	7.9	9.5	
Median	7.0	5.0	5.0	
SD	13.5	11.1	12.4	
Range	0-58	0-44	0-58	
**BOP** (%)				p=0.684
Mean	19.5	17.3	18.5	
Median	15.0	11.0	13.5	
SD	14.1	16.2	14.9	
Range	3-46	0-63	0-63	
**PI** (%)				p=0.485
Mean	30.4	24.3	27.8	
Median	22.0	19.0	20.5	
SD	28.7	19.8	25.0	
Range	3-100	2-69	2-100	

Statistical analysis by two-way ANOVA was used. Statistical significance was determined at $p < 0.05$. Test group= non-surgical treatment with a chloramine as an adjunct. Control group= non-surgical treatment.

Table 4. Twelve-months clinical parameters between the groups

	Test group	Control group	Total group	p-value
**Number of teeth**				
(n)	290	345	635	p=0.179
Mean	19.5	23.0	21.9	
Median	21.0	25.0	24.0	
SD	7.3	6.8	6.2	
Range	2-28	1-29	1-29	
**Number of sites**				
(n)	1160	1380	2540	p=0.179
Mean	77.9	92.0	87.6	
Median	84.0	100	96.0	
SD	29.0	27.1	24.9	
Range	8-112	4-116	4-116	
**Number of PPD ≤ 3 mm** (n/%)	955/82	1170/85	2125/84	p=0.954
Mean	68.2	78.0	73.3	
Median	68.5	87.0	81.0	
SD	25.2	28.9	27.2	
Range	15-97	3-113	3-113	
**Number of PPD 4 mm** (n/%)	82/8	90/7	172/7	p=0.621
Mean	5.9	6.0	5.9	
Median	1.5	5.0	3.0	
SD	8.8	5.3	7.1	
Range	0-28	1-19	0-28	
**Number of PPD 5 mm** (n/%)	52/4	58/4	110/4	p=0.885
Mean	3.7	3.9	3.8	
Median	2.0	2.0	2.0	
SD	4.3	5.8	5.1	
Range	0-14	0-22	0-22	
**Number of PPD ≥ 6 mm** (n/%)	71/6	62/4	133/5	p=0.594
Mean	5.1	4.1	4.6	
Median	2.0	2.0	2.0	
SD	8.5	7.7	8.0	
Range	0-31	0-31	0-31	
**BOP** (%)				p=0.527
Mean	12.9	10.5	11.7	
Median	11.0	8.0	9.5	
SD	11.4	8.9	10.2	
Range	0-33	0-32	0-33	
**PI** (%)				p=0.342
Mean	29.8	21.5	25.6	
Median	25.0	14.0	16.0	
SD	24.3	23.9	24.2	
Range	4-76	0-75	0-76	

Statistical analysis by two-way ANOVA was used. Statistical significance was determined at $p < 0.05$. Test group= non-surgical treatment with a chloramine as an adjunct. Control group= non-surgical treatment.

Table 5. Mean differences in clinical parameters within the test group

	Baseline - Three months	p-value	Baseline - Twelve months	p-value
**Number of teeth** (n)	0.6	p=0.03*	3.8	p=0.023*
**Number of PPD ≤ 3 mm** (n)	-20.1	p=0.000**	-16.1	p=0.015*
**Number of PPD 4 mm** (n)	4.4	p=0.123	4.6	p=0.189
**Number of PPD 5 mm** (n)	4.2	p=0.022*	9.1	p=0.002**
**Number of PPD ≥ 6 mm** (n)	13.9	p=0.000**	17.5	p=0.000**
**BOP** (%) *Mean*	38.1	p=0.000**	45.2	p=0.000**
**PI** (%) *Mean*	15.6	p=0.01	14.1	p=0.1

Test group= non-surgical treatment with a chloramine as an adjunct in the treatment of periodontitis. Statistical significance was determined at p <0.05*. For analysis paired samples t -test was used and adjusted for Bonferroni**.

Table 6. Mean differences in clinical parameters within the control group

	Baseline - Three months	p-value	Baseline - Twelve months	p-value
**Number of teeth** (n)	0.3	0.019*	3.2	p=0.139
**Number of PPD ≤ 3 mm** (n)	-23.3	p=0.00*	-21.9	p=0.001**
**Number of PPD 4 mm** (n)	5.7	p=0.047*	10.1	p=0.002**
**Number of PPD 5 mm** (n)	5.5	p=0.009*	7.5	p=0.002**
**Number of PPD ≥ 6 mm** (n)	13.3	p=0.000**	17.1	p=0.000**
**BOP** (%) *Mean*	28.7	p<0.000**	35.5	p=0.000**
**PI** (%) *Mean*	24.1	p=0.009*	26.7	p=0.003**

Control group= non-surgical treatment in the treatment of periodontitis. Statistical significance was determined at p <0.05*. For analysis paired samples t -test was used and adjusted for Bonferroni**.

Table 7. Prevalence of different items occasionally, fairly often or very often in all patients combined

Conceptual dimension and item	Baseline n (%)	3 months n (%)	12 months n (%)
Functional limitation			
Trouble pronouncing words	4 /38 (11)	5/34 (15)	3/25 (12)
Difficulty chewing	7/37 (19)	3/34 (9)	0 (0)
Food catching	34/38 (89)	24/34 (71)	21/25 (84)
Physical pain			
Sensitive teeth	15/38 (39)	10/34 (29)	10/25 (40)
Sore jaw	21/38 (55)	10/34 (29)	13/25 (52)
Psychological discomfort			
Miserable	7/38 (18)	3/33 (9)	4/25 (16)
Worried	22/38 (58)	16/34 (47)	14/25 (56)
Physical disability			
Avoid eating	5/38 (13)	4/34 (12)	1/25 (4)
Psychological disability			
Depressed	7/38 (18)	6/34 (18)	2/25 (8)
Upset	6/38 (16)	4/33 (12)	8/24 (33)
Social disability			
Irritable with others	8/38 (21)	6/33 (18)	4/25 (16)
Less tolerant of others	6/38 (16)	2/33 (6)	3/25 (12)
Handicap			
Life unsatisfying	5/38 (13)	5/34 (15)	3/25 (12)
Financial loss	11/38 (29)	9/34 (26)	7/25 (28)
