# Supplementary material for: Supra- and sub-gingival instrumentation of periodontitis with the adjunctive treatment of a chloramine – a one-year randomized clinical trial study
Source: Acta Odontol Scand. 2024 Mar 22;83:40288. doi: 10.1080/00016357.2023.2281486 (PMC11302644; doi:10.1080/00016357.2023.2281486)
Supplement: Supra- and sub-gingival instrumentation of periodontitis with the adjunctive treatment of a chloramine – a one-year randomized clinical trial study [file AOS-83-40288-s2.pdf]

Supplementary material has been published as submitted. It has not been copyedited or typeset by Acta Odontologica Scandinavica.

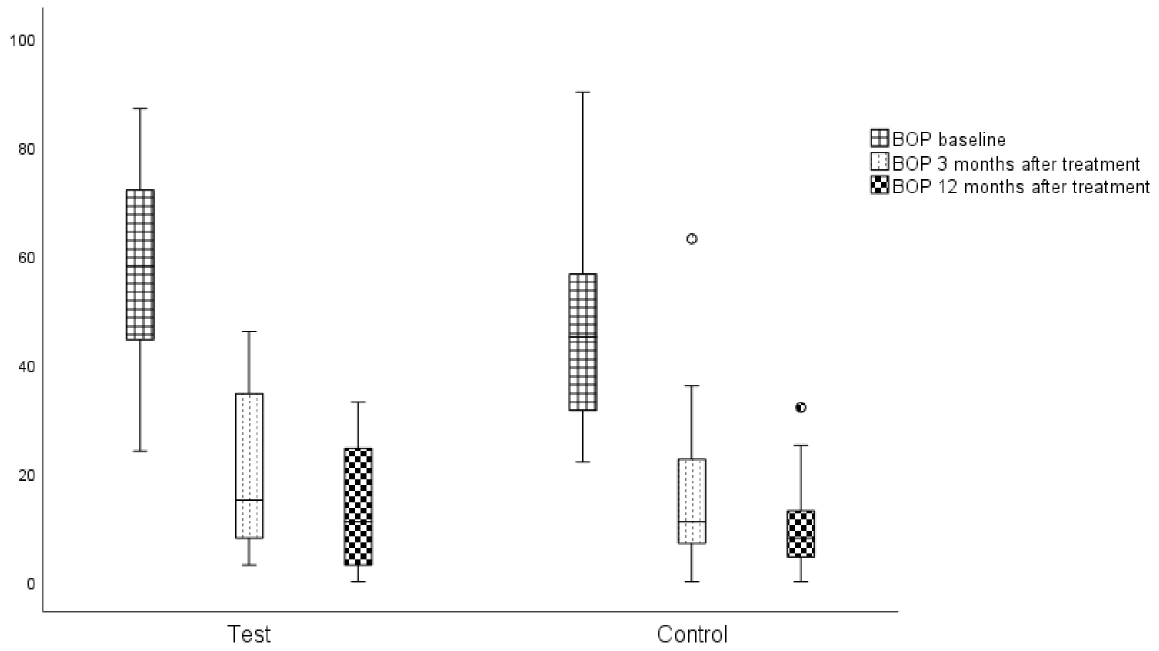

Figure 1. Boxplot of bleeding on probing (BOP) in the test and control group at different time intervals

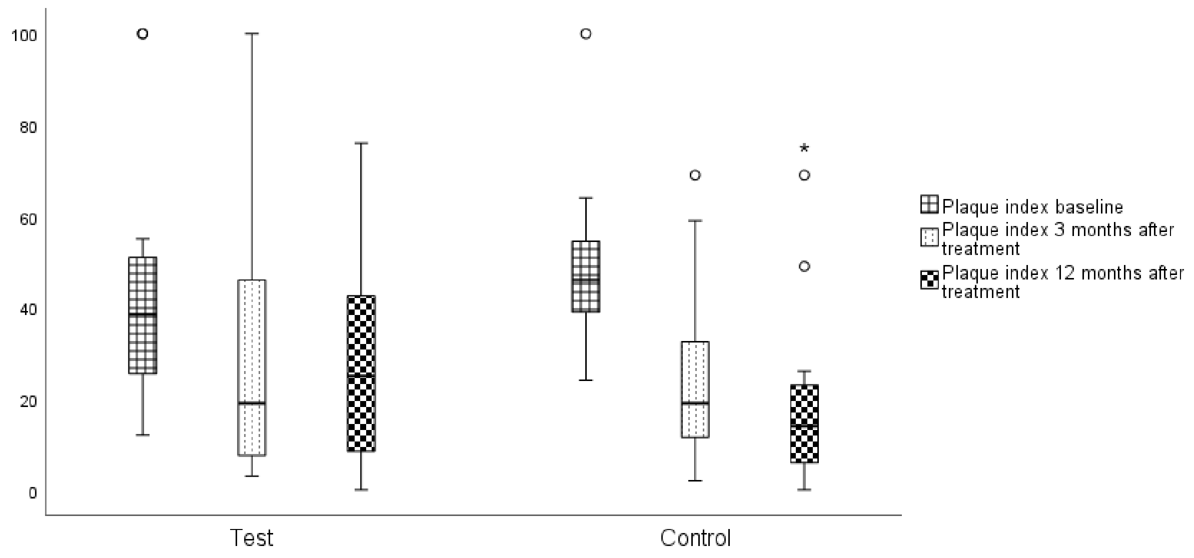

Figure 2. Boxplot of plaque index in the test and control group at different time intervals
